# Do motorcycle helmets reduce road traffic injuries, hospitalizations and mortalities in low and lower-middle income countries in Africa? A systematic review and meta-analysis

**DOI:** 10.1186/s12889-022-13138-4

**Published:** 2022-04-25

**Authors:** Nadifa Abdi, Tara Robertson, Pammla Petrucka, Alexander M. Crizzle

**Affiliations:** 1 School of Public Health, University of Saskatchewan, Saskatoon, Canada; 2 College of Nursing, University of Saskatchewan, Saskatoon, Canada

**Keywords:** Africa, Motorcycle helmets, Hospitalization, Injuries, Motorcycle, Mortality, low- and middle-income countries

## Abstract

**Background:**

Studies in Africa have examined the association between helmet use and injury prevention, however, there has been no systematic review to synthesize the literature within an African context nor has there been any meta-analysis examining the effect of helmet use on injury prevention.

**Methods:**

The review was performed in accordance with the Joanna Briggs Institute for Systematic Reviews. Articles were searched using several databases (e.g. CINAHL, OVID Medline) and select gray literature (e.g. TRID) sources. Articles were included if they were quantitative studies published in English between 2000 and 2019 and examined the association between motorcycle helmet use with head injuries, hospitalizations, and deaths in low- and lower-middle income countries in Africa with comprehensive motorcycle helmet laws. A meta-analysis was performed using pooled effect sizes assessing the impact of helmet use on reducing head injuries.

**Results:**

After screening 491 articles, eight studies met the inclusion criteria. Helmet use ranged from 0 to 43%. The mean age of being involved in a crash was 30 years with males being two times more likely to be involved in motorcycle crashes than females. Drivers (riders) were more likely to be involved in a crash, followed by passengers and then pedestrians. Helmet use reduced injury severity and provided an 88% reduction in serious head injuries (OR 0.118, 95% CI: 0.014–0.968, *p* = 0.049).

**Conclusions:**

In our study, helmet usage significantly reduced the likelihood of fatal head injuries. African countries with no helmet laws should consider adopting helmet use policies to reduce severe head related injuries from motorcycle crashes.

**Supplementary Information:**

The online version contains supplementary material available at 10.1186/s12889-022-13138-4.

## Introduction

Road traffic crashes (RTC) account for a considerable portion of the global public health burden [1] resulting in approximately 1.35 million fatalities and 20 to 50 million injuries annually [2]. RTCs are the 8th leading cause of death in the world and the leading cause of death among those between 5 and 29 years of age [2]. According to the Global Health Burden report, there has been a positive trend over the last 20 years in the reduction of RTCs in high-income countries, yet there is an opposite trend in low- and middle-income countries [3]. In fact, the RTC fatality rates in low-income countries are three times higher compared to high-income countries [4]. The highest RTC fatality rates are reported in Africa with 26.6 deaths per 100,000 people, substantially higher than the 8.3 death per 100,000 people in high-income countries [2, 5]. Consequently, the United Nations (UN) Sustainable Development Goals and the UN Decade of Action on Road Safety are targeting improvements in road safety initiatives in Africa to reduce the number of RTC by 50% in the coming years [5, 6].

Throughout most of Africa, motorcycles are used as both public and private modes of transportation [7, 8]. For example, motorcycles have become increasingly popular over the last decade, due to their ability to navigate through poor road conditions and congested traffic compared to other larger motor vehicles [7, 9]. However, this trend has also resulted in an increase in mortality and morbidity rates [9]. Together, RTCs for motorcyclists, cyclists, and pedestrians account for more than 50% of head-related deaths [2]. Even after controlling for distance travelled, fatalities among motorcyclists and their passengers are approximately 35 times higher than other motor vehicle types [7, 10]. A possible reason for the elevated fatality rate is the lack of protective equipment and shielding [11, 12], such as low helmet use, as evidenced in low and middle-income countries [13, 14].

Studies show the importance of wearing helmets in preventing motorcycle crash (MC) injuries and deaths [2, 15–20]. For example, a Cochrane review found helmet use reduced the risk of head injuries and deaths by 69 and 42%, respectively [14]. Additionally, ecological studies demonstrate motorcycle helmet laws are associated with a decline in morbidity and mortality rates [14, 20, 21]. Although studies have examined the association between helmet use and injury prevention in Africa [19, 22–26], there has been no systematic review to synthesize the literature within an African context nor has there been any study examining the effect of helmet use on injury prevention. Understanding the effectiveness of helmet use on road crashes is a priority area for Safer Africa, an organization funded by Horizon 2020 to improve road safety in Africa [27]. Thus, the objective of this study is to examine the literature on the effectiveness of motorcycle helmet use in reducing the severity of crash related injuries, hospitalizations and mortalities in low to lower-middle income countries in Africa with comprehensive motorcycle helmet laws.

## Methods

### Search strategy

This systematic review was conducted in accordance with the Joanna Briggs Institute (JBI) for Systematic Reviews [28]. A search for published peer-reviewed articles and conference proceedings was performed using the following databases: CINAHL, Public Health Database, Medline OVID, and Web of Science. In addition, a gray literature search was conducted using Transport Research International Documentation (TRID), which combines more than 1.3 million articles from the Transportation Research Board’s Transportation Research Information Services and the OECD’s Joint Transport Research Centre’s International Transport Research Documentation Database. Additionally, we searched for articles using Google Scholar and by manually screening the reference list of eligible articles from the search.

The search terms were developed by two reviewers in consultation with the University of Saskatchewan librarian. The search strategy only included the terms *motorcycles, helmets and Africa* in order to broaden the scope and find more relevant articles. The strategy was developed in Medline and terms were entered in combination using “AND’ and “OR” operators. Terms were then tailored to the other databases used (see Additional file 1: Appendix I).

#### Inclusion and exclusion criteria

The search was limited to low- and lower-middle income countries in Africa, more specifically Ghana, Guinea-Bissau, Kenya, Madagascar, Morocco, Nigeria, Swaziland (Eswatini), and Zimbabwe. The selected countries were identified according to the *Countries with Helmet Laws Meeting Best Practice 2017* from *the WHO Global Status Report on Road Safety 2018* and the *Helmet Laws, Enforcement and Wearing Rates by Country/Area 2015, and* cross-referenced with the World Bank [29]. Countries were selected if they had a comprehensive motorcycle helmet law, defined as a requirement of both drivers and passengers of motorized two-wheelers to wear helmets on all roads, regardless of the engine type [30]. Given this definition, selected countries were required to have the following:

- National motorcycle helmet law
- Applies to drivers and adult passengers
- Applies to all roads
- Applies to all engines
- Helmet fastening required, and standard referred to and/or specified

Peer-reviewed studies and conference proceedings published in English between 2000 and 2019 were included. The date range was determined based on the implementation date of motorcycle helmet regulations, policies, or procedures in the selected countries, which mainly came into effect from the year 2000 onwards. All quantitative study types were included if they measured the impact of helmet use on injuries, hospitalizations, and mortality rates. Motorcycle riders were considered both riders (drivers) and passengers.

Studies were excluded if they were:

- Not in English
- Intervention or Modelling studies
- Qualitative or Evaluation studies (e.g. cost benefit analysis)
- High-income countries or were not the selected countries
- Did not measure the targeted outcomes- hospitalizations, injuries or mortalities
- Did not report on helmet use

Reviewers did not find published articles on motorcycle helmet use in some of the pre-defined countries (i.e. Guinea-Bissau, Madagascar, Morocco, Swaziland, and Zimbabwe). A search was recreated in each database using the same strategy (as detailed above) replacing “Africa” with the individual country name. No additional studies were found.

### Study screening and selection

Following the search, articles were entered into a Microsoft™ Excel sheet; duplicates were removed. Two independent reviewers undertook the screening process which consisted of three phases: title, abstract, and full-text review. In instances where it was unclear whether a study met the inclusion criteria in the title and abstract screening phases, a full-text review was conducted to ensure all relevant studies were captured. There was a 98.7% agreement between the reviewers during the title review stage and 100% agreement during the abstract and full-text review stages. All disagreements were resolved through consensus in the first stage.

**Fig. 1:**
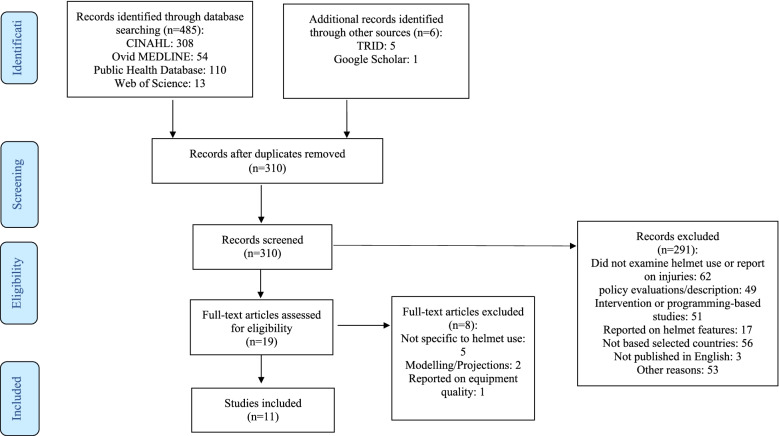
PRISMA Flow Diagram of Study Selection

The study selection process followed the Preferred Reporting Items for Systematic Reviews and Meta-Analyses (PRISMA), illustrated in Fig. 1. The literature search identified a total of 491 results, of which 485 studies were found via database searches, and six studies through grey literature searches; 181 duplicate studies were omitted. Three hundred ten records were screened for title review, resulting in the exclusion of 291 studies: 62 did not examine helmet use or report on injuries; 49 were policy evaluations/description; 51 were intervention or programming-based studies; 17 reported on helmet features (e.g. material); 56 were based in the selected countries; 3 were not published in English; and 53 were for other reasons (e.g. training manual, travel advisory notices; usage of helmets in occupational groups). Full texts were obtained and screened against the inclusion criteria for the remaining 19 records, resulting in the exclusion of eight studies (5 were not specific to helmet use; 2 were removed because they were based on projections/modelling; and one study reported on equipment quality).

### Data extraction

Data were extracted from the included articles using a pinch table (Table 1). Information on the title, author(s), date, and location; study population (i.e. sample size, age, gender, socioeconomic status); study design (inclusion/exclusion criteria), independent variables (including instrument); outcome variable; and results were collected.

**Table 1:** Characteristics of Studies included in Systematic Review

Article (title, author(s), year, and location)	Sample Characteristics	Study Design (inclusion/exclusion criteria)	Independent Variable (including instrument)	Outcome Variable	Results
Sisimwo et al. 2014 [22] Crash characteristics and injury patterns among commercial motorcycle users attending Kitale level IV district hospital, Kenya Kenya	*N* = 384 Mean age of 30.7 years (range 3–80) . 69.8% males; 30.2% females Road Users: riders (45.1%), passengers (38.8%), pedestrians (15.9%) Education: primary school (65.2%), secondary school (31.5%), college (3.3%)	Cross-Sectional Victims of commercial motorcycle crashes at the Crash and Emergency department in Kitale level IV District Hospital in Trans-Nzoia Country Data collected within 24 h of the motorcycle crash	Demographics Crash mechanism, setting, road conditions, collision type, helmet use, road user type Instruments: interviews, questionnaire, patient’s file, medical history, clinical examination	Injury sustained, body region injured, Glasgow Coma Scale (GCS), radiological findings	**Helmet Use and Injuries:** Head Injuries based on Helmet Use for Riders (χ^2^ = 111.35, *p* < 0.001); 37.7% wore helmet; 62.3% did not wear helmet Helmet Users: 1.6% had a head injury and 98% did not Non-Helmet Users: 85.6% had a head injury and 14% did not **Crash setting**: highway (93.9%); rural roads (0.3%). **Crash mechanism**: Motorcycle vs. vehicle (45.6%), Motorcycle vs motorcycle (23.4%), Motorcycle vs. Animal (18.5%), Motorcycle vs. bicycle (9.9%), Motorcycle vs. lone (0.5%), Motorcycle vs. tree/pole (0.5%) **Injury Severity** based on Category of Road Users: Statistically significant (χ^2^ = 129.94, *p* < 0.001) 1. Severe injuries: riders (29.3%); passengers (6.2%) and pedestrians (3.4%) 2. Moderate injuries: riders (63.5%); passengers (88.2%); pedestrians (42.4%) 3. Minor Injuries: riders (7.7%); passengers (5.6%); pedestrians (54.2%) GCS: 64.7% patients with head injury had GSC scores between 9 and 12 (moderate injury); 7.8% were between 3 and 8 (severe injuries) **Hospitalization**: 85.7% treated as in-patients; In those treated as outpatients, 73.1% had minor surgery and 63.8% had major surgery Injuries: 40% head and neck; 39.9% lower body injury; 8.2% chest injury
Sisimwo et al. 2018 [22] Epidemiology of head injuries and helmet use among motorcycle crash injury: a quantitative analysis from a local hospital in Western Kenya Kenya	*N* = 341 Mean age of 31.0 ± 12.9 78.3% males; 21.7% females Road Users: riders (49%), passengers (35%), pedestrians (16%) Education: primary school (62.5%), secondary school (34.9%), tertiary level (2.6%)	Cross-Sectional Victims of commercial motorcycle crashes at the Crash and Emergency department in Kitale level IV District Hospital in Trans-Nzoia Country	Demographics Crash mechanism, helmet use, road user, time of day, day of week, crash location Instruments: Glasgow Coma Scale, interviews, questionnaire, patient’s file, medical history, clinical examination, radiological findings	Injury sustained, type of Injury sustained, injury severity	**Helmet Use**: 28% wore a helmet, 72% did not **Helmet Use and Injuries**: 27% had a head injury and 28% other injuries. Non-Helmet Users: 73% had a head injury and 72% other injuries. Use of helmet was protective of head injury (χ^2^ = 55.78, p < 0.001) Head Injuries by Road Users: riders - head injury (50%), passengers - head injury 50(35%), pedestrian - head injury 22(15%) Being a motorcycle rider was significantly associated with head injuries (χ^2^ = 80.66, p < 0.001) Injuries based on age: 34.6% 20–29 years; 31.7% 30–39 years; 15.5% 10–19 years; 8.5% 40–49 years; 5.6% 50–59 years; 41% > 60 years **Crash Mechanism**: (χ^2^ = 97.97, p < 0.001); motorcycles vs vehicle (48.3%); motorcycles vs motorcycle (22.6%); motorcycles vs pedestrians (17%); motorcycle vs bicycle (9.4%); motorcycle vs animal (1.5%); motorcycle collision with poles and tress (0.6%); motorcycle vs lone (0.6%) **Time of Crash**: 51% afternoon hours (12 pm-5:59 pm), 36.7% morning hours (7 am-11:59 am), 10.3% evening (6 pm-11:59 pm), 2% early morning hours (12 am- 6:59 am) **Day of Crash**: 71.8% between Monday to Friday. Days with the highest number of injuries were Friday (16.1%) and Monday (15.8%)
Mogaka et al. 2011 [31] Factors associated with severity of road traffic injuries, Thika, Kenya Kenya	*N* = 300 *n* = 99 (vulnerable users); *n* = 54 (two-wheeled vehicle users Mean age of 32.4 years (range 3–75) 73% male 27% female Education: none (2%), post-secondary (15%), primary school (49%), secondary school (34%) Road Users: vehicle occupants (68%), two-wheel vehicle users (18%), pedestrians (15%)	Cross-sectional Crash & Emergency Department of Thika District Hospital. Road traffic crash victims attending the hospital within 24 h of the road crash	Demographics Helmet use, road users, day/time of crash, weather Instruments: questionnaires, interviews, clinical information from medical charts; info from police & medical staff	Glasgow Coma Scale, injury severity, body region injured Injury Severity Score (ISS);ISS ≥ 9 (Severe) ISS < 9 (Non-Severe)	**Injury Severity**: Severe Injury 44.6%; Non-Severe Injury 30.3% **Injury Type**:: head & neck superficial injury (60.6%), head & neck laceration (4.0%), head & neck fracture (5.1%), head injury (11.1%), thorax & abdomen superficial injury (7.1%), thorax & abdomen fracture (9.1%), upper extremity soft tissue (13.1%), upper extremity fracture (9.1%), lower extremity soft tissue injuries (48.5%), lower extremity lacerations (4.0%), lower extremity fracture (32.3%) **Crash Mechanism**:: highway crash (80%), weekend crash (57.7%), rainy weather (12%), night time crash (33.3%), angled or head-on collisions (61%)
Saidi & Mutisto, 2013 [24] Motorcycle injuries at a tertiary referral hospital in Kenya: injury patterns and outcome Kenya	*N* = 205 Mean age of 30.8 ± 12.2 years old Male (87.8%) Female (12.2%) Education: primary (41.3%), secondary (39.5%), tertiary (11.6%) Motorcycle road user: riders (67.8%), passengers (16.6%), pedestrians (15.1%)	Cross-sectional All admissions due to motorcycle injuries; data collected from the admissions register of the Crash and Emergency Department at Kenyatta National Hospital Inclusion criteria: motorcycle riders, passengers and pedestrians	Demographics Road user, time of day, helmet use, reason for injury, mode of transport, treatment **Instruments:** Abbreviated Injury Scale, Injury Severity Score, Trauma and Injury Severity Score, Glasgow Coma Scale	Injuries sustained, injury severity, outcome following treatment, length of hospital stay, cost of treatment, resources utilized, mortality at 2 weeks following admission	**Helmet Use**: 43% (50% of riders and 20% of passengers) **Injury Type**: extremities (60.3%), head/neck (32.6%), head injuries (22.7%); femur fractures (18.7%), other lower limb fractures (24.1%), spine (1.97%); visceral (abdominal) injuries (1.97%); chest (0.99%); orbito-facial (3.9%); upper limb fractures/dislocations (3.9%); other injuries (5.4%); 16.3% of patients suffered multi-system injuries. No significant difference in the pattern of predominant injuries sustained by motorcyclists and passengers Head injuries sustained in 37.5% of riders or passengers who did not wear helmets compared to 13.5% in those who did (*p* = 0.049) **Hospitalization**: 62% in hospital two-weeks following admission; 29% discharged; 9% died **Mortality:** based on helmet use: *p* = 0.072; helmet users: 35 alive, 1 dead; non-helmet users: 41 alive; 8 dead **Scene of Crash**: city road (29.6%) and residential areas/suburbs (70.4%) **Day of Crash**: 65% of motorcycle collisions occurred during the day
Oginni et al. 2006 [32] Motorcycle-related maxillofacial injuries among Nigerian intracity road users Nigeria	*N* = 107 Mean age of 29.0 ± 12.5 (range 6–68) Male (78%) Female (22%) Road Users: riders (50.5%), passengers (37.4%), pedestrians (12.1%)	Cross-sectional Two hospitals: patients presenting at the maxillofacial unit following a motor vehicle incident	Demographics Context of crash, road user, helmet use Instrument: questionnaire	Injury type, injury location	**Helmet Use**: 0% **Injury Type**: 48.6% sustained isolated injuries whereas 51.4% had various combinations of injury, abrasion/contusion/hematoma (22.7%), mild laceration (26.9%), moderate laceration (31.9%), through-and-through laceration (11.8%), avulsion (1.7%) **Crash Mechanism**: head-on collision with vehicle (19.6%), head-on collision with other objects (19.6%), rear collision (10.3%), falls (25.2%), collision with motorcycle (10.3%), others (15.0%)
Osifo et al. 2012 [25] Pediatric Road Traffic Accident Deaths Presenting to a Nigerian Referral Center Nigeria	*N* = 143 Mean age of 9.3 ± 5.2 (1–18) Male (67%) Female (33%)	Retrospective cross-sectional Pediatric road traffic crashes admitted to a Nigerian trauma and pediatric surgical center	Demographics Cause of injury, mechanism of injury, helmet use	Injury type, mortality, duration of stay, clinical condition on arrival, resuscitation, treatment	**Helmet Use**: 35.6% wore a helmet; 64.4% did not **Injury Type**: skin laceration/abrasion (35.7%), multiple blunt trauma (27.3%), skull fracture/cerebral injury (11.9), solid visceral rupture (8.4%), fractured bones (10.5%), spinal cord injury (0.7%), lung/heart confusion (5.6%) **Mortality**: Of those using a helmet, 0% died and 35.6% were injured. Of those not wearing a helmet, 10.5% died and 89.5% were injured **Injury User**: 42% were motorcycle users; 63.6% were pedestrians and 36.4% were passengers
Matheka et al. 2015 [33] Road traffic injuries in Kenya: a survey of commercial motorcycle drivers Kenya	*N* = 200 Mean age of 28.4 ± 6.6 Male (98%) Female (2%) Road users: motorcyclists (61.5%), bicyclists (35.5%), auto rickshaw riders (3%)	Cross-sectional survey of commercial motorcycle taxis at 11 sites (convenience sampling) Inclusion criteria: involved in a road traffic crash within the past 3 months	Vehicle type, time of crash, injury type, crash mechanism, safety measures used Instrument: questionnaire	Injuries sustained	**Helmet Use and Other Protective Gear**: 4% wore a helmet only; 44% reported wearing more than one protective gear; 16% wore reflective clothing, 33% did not use any protective equipment Those using protective equipment were 27% less likely to be injured **Injury Type**: Cuts (14.5%), bruises (36%), fracture/dislocations (11%); concussion (1.5%); minor or no injuries (38%) **Time of Crash**: 32.0% occurred during daytime, 22% in the morning, 29% at sunset and 17% at night People injured at night 5x more likely to sustain an injury compared to daytime (OR 5.3, 95% CI 1.7–16.2, *p* < 0.01) **Type of Road**: 36.6% on paved non-highway roads; 31.7% dirt road; 22.0% highway; 8.9% gravel; 0.8% parking lot
Oluwadiya et al. 2014 [34] Vulnerability of motorcycle riders and co-riders to injuries in multi-occupant crashes Nigeria	*N* = 181 Mean age of passengers (29.3) Mean age of riders (32.0) Males: (87%) Females: (13%)	Cross-sectional Patients with motorcycle injuries admitted to the emergency department over a one-year period 125 crashes; 229 patients were injured 37.6% of the crashes involved motorcycles carrying only the rider; 62.4% involved motorcycles with two or more occupants.	Demographics Crash location, road user, number of riders/passengers, helmet use Instrument: hospital intake form, interview with participant, medical records Moderate and severe injuries were defined as ISS of 9–15 and ISS =/> 16, respectively	Injuries sustained, injury severity	**Helmet use**: Rider: Helmet 9.1%; Non-Helmet 90.9% Co-Rider: Helmet 9.4%; Non-Helmet 90.6% **Injury Prevalence**: 69.6% sustained injuries; 30.4% did not. Those with injuries were riders (42.1%) and co-riders (57.9%) Of the 78 crashes involving 2 or more motorcycle occupants: 53.8% caused injuries to riders and passengers together 30.8% caused injuries to passengers alone 15.4% caused injuries to riders alone A significantly higher percentage of females (*p* = .045) were also injured on > 2-occupant motorcycles (19.7%) compared to 2-occupant motorcycles (12.2%) **Injury Location**: Head (*n* = 36): Rider: 30.5%; Co-rider: 69.5% Face (n = 34): Rider: 50.0%; Co-rider: 50.0% Chest (n = 8): Rider: 62.5%; Co-rider: 37.5% Abdomen (n = 2): Rider: 50.0%; Co-rider: 50.0% Extremities (*n* = 80): Rider: 31.3%; Co-rider: 68.7% External (*n* = 41): Rider: 53.7%; Co-rider: 46.3% **Injury Severity**: moderate injury (co-riders 90.4%; riders 100%) severe injury (co-riders 9.6%; riders 0%)

### Data synthesis

#### Critical appraisal

The JBI Critical Appraisal checklists were used to assess the validity, methodological quality and bias in each study. The nine-question Checklist for Prevalence Studies [35] was used to assess six studies classified as descriptive by reviewers as they characterized the prevalence of the exposure (i.e. helmet or non-helmet use) and outcome (i.e. injuries and/or death). The eight-question Checklist for Analytical Cross-Sectional Studies [36] was used for the other five studies categorized as analytical as they examined the relationship between the exposure and outcome. Two reviewers independently conducted the critical appraisals for each study with an 88% consensus. Disagreements were resolved by the inclusion of a third reviewer. Three studies were excluded from the review from being classified as being of low methodological quality. In this review, low methodological quality referred to failing more than half of the criteria (50%). Eight studies met the criteria and were considered as being of moderate quality.

As shown in Table 2, three of the six descriptive studies met the criteria for inclusion [31–33]. One article met seven out of the nine criteria [31] while the other two studies met five of the nine criteria [32, 33].

**Table 2:** Joanne Briggs Institute Critical Appraisal of Prevalence Studies

Criteria	Factors associated with severity of road traffic injuries (Mogaka et al. 2011 [31])	Motorcycle injuries in a developing country and the vulnerability of riders, passengers, and pedestrians (Solagberu et al. 2006 [19])	Motorcycle injuries in North-Central Nigeria (Nwadiaro et al. 2011 [8])	Motorcycle-related maxillofacial injuries among Nigerian intracity road users (Oginni et al. 2006 [32])	Patterns of morbidity and mortality amongst motorcycle riders and their passengers in Benin-City Nigeria: one-year review (Nzegwu et al. 2008 [37])	Road traffic injuries in Kenya: a survey of commercial motorcycle drivers (Matheka et al. 2015 [33])
1. Was the sample frame appropriate to address the target population?	Yes	No	No	No	No	No
2. Were study participants recruited in an appropriate way?	No	No	No	Yes	No	No
3. Was the sample size adequate?	No	No	No	No	No	Unclear
4. Were the study subjects and setting described in detail?	Yes	Yes	Yes	Yes	Yes	Yes
5. Was data analysis conducted with sufficient coverage of the identified sample?	Yes	No	Unclear	No	No	Yes
6. Were valid methods used for the identification of the condition?	Yes	Yes	Yes	Yes	Yes	No
7. Was the condition measured in a standard, reliable way for all participants?	Yes	Yes	Unclear	Yes	Unclear	Yes
8. Was there appropriate statistical analysis?	Yes	Unclear	Yes	Yes	Unclear	Yes
9. Was the response rate adequate, and if not, was the low response ate managed appropriately?	Yes	Yes	Unclear	Unclear	Unclear	Yes
Overall Rating	7 out of 9	4 out of 9	3 out of 9	5 out of 9	2 out of 9	5 out of 9
Overall Appraisal	Include	Exclude	Exclude	Include	Exclude	Include

As shown in Table 3, all five analytical studies passed the methodological appraisal review [22, 23, 25, 32, 34]. One study met seven out of the eight criteria [22]. Three studies met six out of the eight criteria [23, 25, 34]. Inclusion criteria were clearly defined in all studies, and the exposures were measured in a valid and reliable way. Only one study mentioned and adjusted for confounders [22].

**Table 3:** Joanne Briggs Institute Critical Appraisal of Prevalence Studies

Criteria	Crash characteristics and injury patterns among commercial motorcycle users attending Kitale level IV district hospital, Kenya (Sisimwo et al. 2014 [22])	Epidemiology of head injuries and helmet use among motorcycle crash injury: a quantitative analysis from a local hospital in Western Kenya (Sisimwo et al. 2018 [23])	Motorcycle injuries at a tertiary referral hospital in Kenya: injury patterns and outcome (Oginni et al. 2006 [32])	Pediatric Road Traffic Accident Deaths Presenting to a Nigerian Referral Center (Osifo et al. 2012 [25])	Vulnerability of motorcycle riders and co-riders to injuries in multi-occupant crashes (Oluwadiya et al. 2014) [34]
1. Were the criteria for inclusion in the sample clearly defined?	Yes	Yes	Yes	Yes	Yes
2. Were the study subjects and the setting described in detail?	Yes	Yes	Yes	Yes	Yes
3. Was the exposure measure in a valid and reliable way?	Yes	Yes	Yes	Yes	Yes
4. Were objective, standard criteria used for measurement of the condition?	Yes	Yes	Yes	Yes	Yes
5. Were confounding factors identified?	Yes	No	No	No	No
6. Were strategies to deal with confounding factors stated?	Yes	No	No	No	No
7. Were the outcomes measured in a valid and reliable way?	Unclear	Yes	Yes	Unclear	Yes
8. Was appropriate statistical analysis used?	Yes	Yes	Yes	Yes	Yes
Overall Rating	7 out of 8	6 out of 8	6 out of 8	5 out of 8	6 out of 8
Overall Appraisal	Include	Include	Include	Include	Include

#### Preliminary assessment and Meta-analysis

A preliminary assessment was conducted to determine whether a meta-analysis was appropriate and which studies would be eligible to be included. The PICO (population, intervention, comparator, outcome) method was utilized and documented in Table 4. A meta-analysis was performed on three studies measuring head injuries as an outcome, examining helmet use as the intervention. Effect size was reported as an odds ratio (OR), with a 95% confidence interval (CI) and corresponding *p*-value. A random effects model was applied due to the distribution of true effect sizes amongst the three studies. Heterogeneity was analyzed using I squared (I^2^). Funnel plot and Egger test were the indicators used to assess publication bias. All analyses including sensitivity analysis were performed using the Comprehensive Meta-Analysis V3 software.

**Table 4:** PICO Analysis of Included Studies

Study	Design	Population	Comparator						Outcome							
			Helmet use distribution	Helmet use by road user	Time of crash	Day of crash	Crash setting	Crash method	Injury severity by road user	Injury type by road users	Head injuries using helmets	Mortality using helmets	Mortality by injury severity	Mortality by road user type	Level of Consciousness	Injury type
Sisimwo et al., 2014 [22]	Analytical	MCC victims at A&E (hospital)	Y	N`	N	N	Y	Y	Y	N	Y	N	N	N	Y	N
Sisimwo & Onchiri, 2018 [23]	Analytical	MCC victims at A&E (hospital)	Y	N	Y	Y	N	Y	N	Y	Y	N	N	N	N	N
Saidi & Mutisto, 2013 [24]	Analytical	MCC victims at A&E (hospital)	Y	Y	Y	N	Y	N	N	N	Y	Y	Y	N	N	Y
Osifo et al., 2012 [25]	Analytical	Pediatric RTA victims presenting at a trauma center (includes MCC victims)	Y	N	N	N	N	N	N	N	N	Y	N	Y	N	N
Oluwadiya et al., 2014 [34]	Analytical	MCC victims at A&E (hospital)	Y	Y	N	N	N	N	Y	Y	N	N	N	N	N	Y
Osoro et al., 2011 [38]	Prevalence	RTA victims presenting at the hospital (includes MCC victims)	N	N	N	N	N	N	N	N	N	N	N	N	N	Y
Oginni et al., 2006 [32]	Prevalence	MCC victims at maxillofacial unit (hospital)	Y	N	N	N	N	Y	N	N	N	N	N	N	N	Y
Matheka et al., 2015 [33]	Prevalence	MCC victims within preceding 3 months of survey	Y	N	Y	N	Y	N	N	N	N	N	N	N	N	Y

## Results

### Design and setting

All eight studies were cross-sectional and used convenience sampling. Seven of the eight studies were conducted prospectively; one was retrospective. Five studies were conducted in Kenya and three in Nigeria. Motorcycle crashes were the variable of interest for six studies while two studies looked at RTCs including motorcycle-related crashes. Participants were recruited from hospitals in seven studies; five recruited victims involved in motorcycle crashes and two studies examined victims of road related traffic crashes. The settings included the Crash and Emergency department in five studies, one maxillofacial unit, and one referral trauma and pediatric surgical center. One study collected data from participants who were previously involved in MCCs using a structured questionnaire in eleven rural and urban sites in Kenya. Three studies were conducted over a one-year period, three took place in less than a year, and two were conducted for more than a year.

### Population characteristics

The sample sizes varied between 107 and 384 patients. MCCs accounted for 18 to 53% of all crashes among the studies. Among all studies, the study population consisted of more males than females, with approximately a 2:1 ratio in three of the studies; 3:1 in two studies; and more than 3:1 in three studies. The mean age for nearly all the studies was about 30 years old, however, some age and gender differences emerged. One study found the peak age of sustaining motorcycle related injuries for males was 20–29 years compared to the 10–19 age bracket for females [32]. Another study found that the average age of male drivers who suffered injuries was 25–31 years, followed by 18–24, with the opposite being observed for females [33]. However, one study reported no difference between male and female MCC injury victims [23]. More than half of the studies classified participants based on road type user, in which three differentiated between riders, passengers, and pedestrians; one study assessed riders and passengers; and one of the RTC studies distinguished between two-wheeled vehicle occupants and pedestrians. The road user most injured were riders, followed by passengers and pedestrians, respectively, across all studies. In the four studies that assessed education level, primary school (41–65%) was the highest level attained by the participants, followed by secondary level (32–40%), then college/tertiary level (2.6–15%).

### Helmet use

Six studies reported helmet use at the time of a crash ranged from 0 to 43%. Only one study reported that none of the crash victims wore a helmet at the time of a crash [32]. Two studies compared helmet use between road users [24, 34]. Helmet use ranged from 9 to 50% for riders and 9–20% for passengers. One study found that people on 2-occupant motorcycles were more than seven times more likely to wear helmets compared to more than 2-occupants riders [34].

### Time and day of crash

Three studies compared time and day of crash. One study found that 51% of the crashes occurred during the afternoon hours (7 am-11:59 am), followed by the 36.7% in the morning (7 am-11:59 am), 10.3% in the evening (6 pm-11:59 pm) and 2.1% in the early morning (12 am-6:59 am), however, time of crash was not significantly associated with head injuries [23]. Similarly, another study found that 32% of crashes occurred during the day, 22% in the morning, 29% at sunset and 17% at night (hours of day undefined) [32]. Individuals involved in crashes during the night were five times more likely to suffer injuries compared to daytime crashes (Unadjusted OR 5.3, 95% CI 1.7–16.2, *p* = 0.00) [33].

About 65% of motorcycle crashes occurred during the day (versus night) [24] and almost three-quarters (71.8%) of head-related motorcycle crashes occurred on weekdays (Monday to Friday) compared to the week-end [23]. The highest proportion of crashes occurred on Friday’s (16.1%) and Mondays (15.8%) respectively, although there was no association between head injuries and the days of the week the crash occurred [22].

### Setting of crash

The studies examining crash settings were varied [22, 24, 32] One study reported that 93.9% all the crashes occurred on the highway and 0.3% occurred on rural roads [22] whereas another study reported that 70.4% took place on smaller roads in residential areas and the suburbs compared to the 29.6% that occurred on main city roads [24]. In another study, 36.6% of the injuries occurred on paved non-highway roads, 31.7% on dirt roads, 22.0% on the highway, 8.9% on gravel and 0.8% in the parking lot [33].

### Mechanism of crash

Three studies assessed mechanism of crash. Motorcycle-vehicle collisions accounted for 45.6% of the crashes, followed by 23.4% of motorcycle-motorcycle collisions, 18.5% of motorcycle-animal collisions, 9.9% motorcycle-bicycle, along with motorcycle-lone and motorcycle tree/pole collisions, each representing 0.5% of the crashes [22]. In a later study by the same first author, 48.3% of collisions were motorcycle-vehicle, followed by 22.6% motorcycles-motorcycle crashes, 17% motorcycles-pedestrians, 9.4% motorcycle-bicycle, 1.5% motorcycle-animal, and 0.6% motorcycle-poles/trees crashes [23]. Similarly, another study reported the nature of the collisions were motorcycle to vehicle (19.6%), tied with head-on collision with other objects (19.6%), rear collisions (10.3%), falls (25.2%), collision with motorcycle (10.3%) and others (15.0%) [32].

### Injury type

Head and neck injuries ranged from 40 to 60.6% [22, 31], followed by lower extremities injuries that ranged from of 39.9% [22] to 48.5% [31]. There was a significant reduction in head injuries in those wearing helmets in three studies. Head injuries in those wearing helmets ranged from 1.6 to 37.7% compared to 62.3 to 85.6% in riders not wearing helmets [22, 23, 33]. Extremities were the main site of injury in two studies, followed by head or neck injuries [24, 34]. Injury types varied in the three studies with one study reporting skin lacerations and abrasions being the primary injury type for all road traffic crash victims including motorcycle crashes [25]. One study reported minor injuries made up 38% of injury types followed by bruises (36%) [33]. A study analyzing maxillofacial injuries found moderate laceration as the main type of soft tissue injury and the mandible as the leading type of fracture [32]. The proportion of injuries were significantly reduced in riders using helmets although these studies did not specify the exact nature of the injuries. For example, one study found that 28 and 35.6% of helmet users were injured compared to 72 and 89.5%, respectively [21, 33].

Two studies examined injuries sustained by road users. One study reported that 50% of riders suffered head injuries compared to 35% of passengers and 22% of pedestrians [22]. Riders (49%) also sustained other injuries (not specified) compared to 35% of passengers and 16% of pedestrians [22]. Being a rider was significantly associated with sustaining a head injury (χ^2^ = 80.658, *p* < 0.00) [23]. The other study observed that more passengers suffered head injuries (69.5%) and injuries to the extremities (68.7%) compared to 30.5% of riders that sustained head injuries and 31.3% suffered injuries to the extremities [34]. Alternatively, riders sustained more chest (62.5%) and external (53.7%) injuries compared to chest (37.5%) and external injuries (46.3%) of passengers [28]. However, an equal percent of riders and passengers sustained facial (50%) and abdominal (50%) injuries [34].

### Injury severity based on type of road user

Many studies used instruments to measure patterns of morbidity and mortality. The Glasgow Comma Scale (GCS) was used to measure head injury and severity in four studies [22–24, 31]; and the Abbreviated Injury Scale (AIS) was used in one study [24]. In three studies, the Injury Severity Score (ISS) was used to measure severity [24, 31, 34]; and one study used the Trauma and Injury Severity Score (TRISS) to measure the probability of survival [24]. Questionnaires, interviews or a combination of the two were used in six studies [22, 23, 31–34]. Information from patient files, admission register books, or medical charts were collected in 6 studies [22, 23, 25, 32–34]. Other instruments utilized included clinical examination [22, 23] and radiological data [22, 23], as well as information from the police and healthcare professionals [31].

Two articles explored the relationship between type of road users and injury severity. One study reported that 69.5% of road users suffered moderate injuries, 16.1% severe injuries and 14.2% minor injuries [22]. More riders (29.3%) suffered severe injuries compared to passengers (6.2%) and pedestrians (3.4%) [22]. Alternatively, passengers sustained more moderate injuries (88.2%) compared to riders (63.5%) and pedestrians (42.4%) [22]. More pedestrians (54.2%) sustained minor injuries compared to riders (7.7%) and passengers (5.6%) [22]. The relationship between injury severity and road user was found to be statistically significant (χ^2^ = 129.94, *p* < 0.001) [22]. Another study reported that 100% of the riders suffered moderate injuries while 90.4% of passengers sustained moderate injuries and 9.6% suffered severe injuries [34].

### Mortality

The relationship between helmet use and mortality was examined in two studies. One study examined predictors of mortality at 2 weeks after a motorcycle crash, in which 2.8% of those who used helmets died compared to 14.3% of non-helmet users [24]. Injury severity was predictive of mortality within 2 weeks of admission [24]. In another study, none of the 21 patients that wore a helmet at the time of crash died compared to 10.5% that did not wear a helmet [25].

## Meta-analysis

### Head injury

**Fig. 2:**
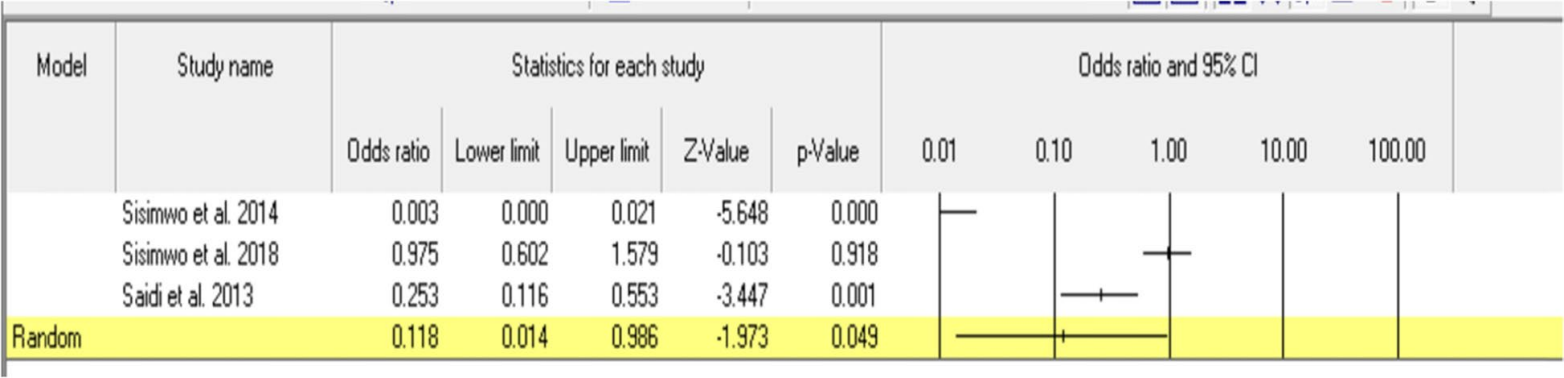
Random effects meta-analysis comparing helmet use vs non-helmet use. Reference point was non-helmet use (OR = 1.0)

Figure 2 displays the random effects meta-analysis results. Three studies measuring effects of helmet use on head injuries, compared to non-helmet use, were included in the meta-analysis. Overall, the pooled results were statistically significant and indicated that helmet use provides an 88% reduction in sustaining head injuries (OR = 0.118, 95% CI: 0.014 to 0.968). In two of the individual studies, there was a statistically significant protective association between helmet use and head injury [22, 24]. In one study, there was no protective effect of using helmets and sustaining a head injury [23].

Substantial heterogeneity (I^2^ = 94.256) was noted among effect sizes which may be attributed to confounders as only one study adjusted for them [22] with the other two not identifying confounders or failing to report them in their study [23, 24]. Egger’s regression test was significant for publication bias (*p* = 0.049).

## Discussion

The findings indicate a low prevalence of motorcycle helmet use ranging from 0 to 43%. Helmet use ranged from 28 to 43% in Kenya [22–24] and 0–35.6% in Nigeria [25, 32, 34]. This is consistent with prior findings in other low- and lower-middle income countries in Africa; however, they are different from non-African low- and low-middle income countries. For instance, in a cross-sectional observational study in Ghana, the prevalence of helmet use was 45.8% in riders and 3.7% in passengers [26]. In India, helmet use was observed to be 89% in Calicut city but only 23% in rural areas [39]. Greater use of helmets in urban centres was credited to stringent and consistent enforcement strategies that were not found in the rural areas [39]. Helmet use in an Ethiopian study was predicted by having a valid license, having greater driving experience, driving greater distances, being exposed to accidents and having an accident risk perception [40]. However, a comprehensive helmet law was not a motivator to wearing a helmet which may explain why only 12.4% of riders wore a helmet [40]. In high income countries such as the USA, 99% of motorcyclists wear helmets in states that have helmet laws compared to 71% that do not. Moreover, 89% of motorcyclists in states with helmet laws were compliant with helmet safety regulations where only 56% were compliant in states without helmet laws [41]. Considering the trend of low helmet use in our study, and in low-middle income countries [26, 39, 40, 42], efforts are needed to examine underlying factors such as lack of enforcement strategies, or uninformed knowledge, attitudes, and practices of riders.

The findings that helmet use reduced injuries was supported by the meta-analysis. Our meta-analysis found that wearing a helmet at the time of the crash was protective against head injuries. Our study also found that helmet use reduced mortality consistent with prior studies in developed [15–20] and developing countries [14, 42, 43]. For example, studies from Kenya found a risk reduction of head injuries by 69% and mortality by 42% from using helmets [14, 43] and another study in Vietnam found the implementation of helmet laws in Cu Chi city resulted in a 65% decrease in head injuries and a 31% reduction in the number of deaths from motor vehicle crashes [42]. It is clear that helmet use not only reduces the likelihood of significant injuries but also saves the health care system in treatment and rehabilitation costs [44].

Our study also found that riders were more likely to be involved in MCCs (range 45–68%) compared to passengers (range 17–39%), which is consistent with other African studies not included in this review. For example, in Benin-City, Nigeria, 60.8% of riders were involved in MCCs compared to 39.2% of passengers [37]. Our review also found that approximately 15% of crashes occur in pedestrians. This could be because there is no designated sidewalk for pedestrians (resulting in them walking along the road) and poor street lighting, increasing the risk of being struck by a motor vehicle [23]. Future research should consider differences between riders, passengers and pedestrians on several criteria, including age distribution, education level, rate of helmet use, head injury sustained, other types of injuries sustained, injury severity, and mortality. Additionally, there may be differences in helmet practices for those that use motorcycles for employment vs personal use. For example, a study in Cameroon found that commercial motorcycle riders were 4x less likely to wear any protective gear (including helmets) compared to riders who used motorcycles for personal use [45]. Distinctions between the category of road users can reveal trends or patterns that may be useful in tailoring interventions.

Our review shows that males were at least two times as likely to be admitted to a hospital following a motor vehicle crash. Other findings have demonstrated that males are also more likely to be involved in MCCs and RTCs, which reflects that males are typically more likely to engage in risk taking behaviors including speeding [7, 46]. Among all the studies in this review, primary school was more often the highest level of education obtained which may contribute to difficulty navigating road signs or understanding the rules of the road. Additionally, our review found a trend that crashes occur in the daytime and during the week (compared to the week-end). It is possible that motorcycle crashes during the day are related to employment, however, none of the included studies captured the reasons why crashes occurred or the circumstances that led up to the crash. Understanding the factors associated with crashes can result in infrastructure changes to reduce crash risk, especially in areas where traffic is dense.

## Limitations and opportunities for further research

This review also found several methodological gaps that can be improved upon in future research. First, the studies identified for the systematic review and meta-analysis were all cross-sectional which only examined the association between exposure and outcome variables at one point in time [47], precluding the ability to determine causality [48–50]. Additionally, only one of the three studies included in the meta-analysis adjusted for confounding factors which could explain the high heterogeneity observed. There may also have been differences in standards of helmets, or the type of helmets used (i.e. full-face or half face) since these were not defined or captured in the studies. The use of non-standard helmets that are often used in low- and middle-income countries may negate the positive benefits of having a comprehensive helmet law [51]. Given the price of a standard helmet is 2-3x greater than that of non-standard helmets [50], future studies should assess the types of helmets used (i.e. full-face versus half-face; standard versus novelty helmets) and examine practices such as proper fastening of the helmet to garner a more in-depth understanding of this issue. There is also a need to determine how many riders and/or passengers use helmets. According to the African Road Safety Action Plan, fewer than 18% of African countries provide information on rates of helmet use [52].

All studies employed convenience sampling which does not capture a true representation of the general population [53]. While most studies recruited participants from hospitals, studies did not include those who did not present at the hospital due to minor injuries or those pronounced dead at scene [54]. Consequently, it is possible the true effects of helmet use on reducing injuries is understated [55]. Other limitations included the lack of published helmet use studies from African countries with comprehensive helmet policies, specifically Guinea-Bissau, Madagascar, Morocco, Swaziland, and Zimbabwe. Capturing data from these countries would reaffirm the trend surrounding helmet use in Africa. Data from Africa are generally underreported and there are many inconsistencies between data that is collected, due to lack of road traffic data collection systems [56–58].

Research has demonstrated the impact of motorcycle helmet legislation in improving helmet uptake, however, governance alone is insufficient [43, 59–64]. While the reviewed studies did not provide details on how helmet laws were enforced or advertised, it is clear that helmet uptake is important in order to reduce the number of RTC injuries and fatalities. An examination of enforcement strategies, stigma, and advertising campaigns is warranted in African settings, including differences between urban and rural contexts. Lessons learned from developed countries in implementing helmet laws have potential to be applied and tailored for African settings.

## Conclusion

The outcomes of this systematic review support the assumption that helmet use protects against head injuries, according to the current literature addressing the case of African riders. Therefore, low- and middle-income countries in Africa should highly consider implementing comprehensive motorcycle helmet laws. Further research efforts are crucial in these countries due to the high prevalence of crashes resulting in head injuries. Evidence-based data and collaborative efforts between stakeholders are required to inform the development of helmet policies to improve road safety in low and lower-middle income countries.

## Supplementary Information

**Additional file 1: Appendix I.** Search Strategy.

## Data Availability

The search strategy including the databases used and the key terms are available upon request from the corresponding author.

## Abbreviations

RTC: Road Traffic Crashes
MCC: Motorcycle accident
JBI: Joanna Briggs Institute
OR: Odds ratio
CI: Confidence interval
I^2^: I-squared
GCS: Glasgow Comma Scale
AIS: Abbreviated Injury Scale
ISS: Injury Severity Score
TRISS: Trauma and Injury Severity Score
