# Supplementary material for: Do motorcycle helmets reduce road traffic injuries, hospitalizations and mortalities in low and lower-middle income countries in Africa? A systematic review and meta-analysis
Source: BMC Public Health. 2022 Apr 25;22:824. doi: 10.1186/s12889-022-13138-4 (PMC9036710; doi:10.1186/s12889-022-13138-4)
Supplement: Supplementary file 1 — Additional file 1: Appendix I. Search Strategy. [file 12889_2022_13138_MOESM1_ESM.docx]

**APPENDIX I – Search Strategy**

The search terms were developed by two reviewers in consultation with the University of Saskatchewan Librarian (VD). A pilot phase was undertaken to test which key terms and combinations would yield an appropriate amount of results. The initial search strategy generated an excessive amount of results (e.g. 120,000 + studies) using the following terms which were entered in combination, using “AND” and “OR” operators. Another search was conducted to include “Africa”.

1. *(injuries OR injury OR wounds OR hospitalization OR accidents OR motor vehicle collisions OR mortality OR fatality OR death) AND (motorcycles OR motorcycle) AND (head protective devices OR helmet OR helmets) AND (low-middle income countries)*
2. [*(motorcycle OR motorcycles) AND (helmet OR helmets OR head protective devices) AND (injuries OR fatalities OR wounds OR death OR hospitalization OR mortality OR accidents) AND Africa AND low-income countries)*](https://search-proquest-com.cyber.usask.ca/myresearch/savedsearches.checkdbssearchlink:rerunsearch/1600809/SavedSearches?site=publichealth&t:ac=SavedSearches)

The search term strategy subsequently went through two more revisions using Medline and Public Health Database as shown below. The first strategy substituted “low-middle income countries” with the individual country names that were combined in one search using “OR” and with the outcomes of interest, but it did not generate any results. The second strategy replaced individual country names with “Africa” and only included the outcome of interest to “injuries and fatalities”. This generated 5 results in Medline and 85 results in the Public Health Database:

1. [*(motorcycle OR motorcycles) AND (helmet OR helmets OR head protective devices) AND (injuries OR fatalities OR wounds OR death OR hospitalization OR mortality OR accidents OR road safety) AND (Africa OR Ghana OR Guinea-Bissau OR Eswatini OR Swaziland OR Kenya OR Madagascar OR Morocco OR Nigeria OR Zimbabwe)*](https://search-proquest-com.cyber.usask.ca/myresearch/savedsearches.checkdbssearchlink:rerunsearch/1600936/SavedSearches?site=publichealth&t:ac=SavedSearches)
2. *(motorcycle) AND (helmet) AND (injuries OR fatalities) AND (Africa)*

The final search strategy was developed in Medline to refine the search terms and broaden the scope to find more relevant articles. These terms were entered in combination as shown below:

*(motorcycles OR motorcycle) AND (head protective devices OR helmet OR helmets) AND (Africa)*
